# The anti-mesothelin monoclonal antibody amatuximab enhances the anti-tumor effect of gemcitabine against mesothelin-high expressing pancreatic cancer cells in a peritoneal metastasis mouse model

**DOI:** 10.18632/oncotarget.26117

**Published:** 2018-09-18

**Authors:** Tatsuzo Mizukami, Hirofumi Kamachi, Yuki Fujii, Fumihiko Matsuzawa, Takahiro Einama, Futoshi Kawamata, Nozomi Kobayashi, Yutaka Hatanaka, Akinobu Taketomi

**Affiliations:** ^1^ Department of Gastroenterological Surgery I, Hokkaido University Graduate School of Medicine, Kita-Ku, Sapporo, Hokkaido, 060-8638, Japan; ^2^ Research division of Companion Diagnostics, Hokkaido University Hospital, Kita-ku, Sapporo, Hokkaido, 060-8638, Japan

**Keywords:** amatuximab, mesothelin, pancreatic cancer, peritoneal metastasis, adjuvant chemotherapy

## Abstract

Pancreatic cancer often has a very poor prognosis, even after complete resection. The recurrence of hepatic and peritoneal metastases is an important prognostic factor; therefore, the development of improved adjuvant therapy is urgently required. Mesothelin is a cell surface glycoprotein whose expression is restricted to a variety of cancer types, including pancreatic cancer. This expression pattern makes mesothelin an attractive target for cancer therapy, and several agents targeting mesothelin are currently in clinical trials. Here, we used the chimerized high-affinity anti-mesothelin monoclonal antibody amatuximab to investigate its effect on peritoneal metastasis. We used the AsPC-1 pancreatic cancer cell line engineered to express Gaussia luciferase (Gluc), (AsPC-1-Gluc) for *in vivo* experiments. Results showed that while amatuximab was not directly cytotoxic on an AsPC-1-Gluc tumor cells in a peritoneal metastasis model, it prevented the formation of tumor growth. In combination therapy with gemcitabine, amatuximab exhibited synergistic killing. Our results suggest that blockade of mesothelin by amatuximab may be a useful strategy for preventing the peritoneal dissemination of pancreatic cancer under an adjuvant setting.

## INTRODUCTION

Pancreatic cancer is the fourth leading cause of cancer death in the United States, and its 5-year survival rate is only 6% [1]. Surgical resection remains the only potentially curative therapeutic option. However, pancreatic cancer proceeds asymptomatically in many cases, and surgical resection is feasible in only 10%–20% of patients at the time of initial diagnosis [2]. Even after complete resection, the long-term survival rate remains very poor [3, 4]. Hepatic and peritoneal metastasis were reported to be important prognosticators in pancreatic cancer [5]. Microscopically curative resection of pancreatic carcinoma revealed frequent micrometastatic dissemination, which is not detected by imaging or gross pathology in cases of radical treatment [6]. Therefore, controlling the microdissemination of residual malignant cells postoperation is critical for improving prognosis.

Currently, gemcitabine (GEM) [7] or S-1 [8] adjuvant treatment has been shown to improve patient prognosis after surgical resection, but the prognosis is still worse compared with other gastrointestinal cancers. Therefore, the development of improved postoperative therapy is urgently required.

Mesothelin is a 40-kDa cell surface glycoprotein and its expression in normal human tissues is limited to mesothelial cells lining the pleura, pericardium and peritoneum [9, 10]. Moreover, mesothelin is highly expressed in various types of cancers, including malignant mesothelioma [11], ovarian cancer [12] and pancreatic cancer [13–15]. The full length of human mesothelin gene encodes a 71-kDa precursor protein, which is physiologically cleaved by furin-like proteases into a 40-kDa C-terminal fragment that remains membrane bound, and a 31-kDa N-terminal fragment, which is secreted into the blood [10]. The C-terminal, membrane-bound 40-kDa fragment is named mesothelin and is attached to the cell membrane through a glycosyl-phosphatidylinositol (GPI) anchor [10]. This expression pattern makes mesothelin an attractive target for cancer therapy and several agents targeting mesothelin are currently in clinical trials [16–18]. Amatuximab is, a chimerized high-affinity monoclonal IgG1/k antibody targeting mesothelin [19]. It has been shown to be able to inhibit the heterotypic cell adhesion of mesothelin-positive tumor cells to CA125-expressing tumor cells as well as kill mesothelin-positive tumor cells via antibody dependent cellular cytotoxicity. Based on its safety profile in a Phase I study and preclinical studies showing synergy with chemotherapy, amatuximab was combined with GEM in a Phase II study in patients with previously untreated unresectable stage 3 or 4 pancreatic cancer. This study (NCT00570713) did not demonstrate improved efficacy over chemotherapy alone. Subsequent tumor imaging human trials using a radio-labeled amatuximab demonstrated that amatuximab was unable to penetrate pancreatic tumors in contrast to less mucus-producing and more vascular tumors such as mesothelioma [20].

In light of these data, the utility of amatuximab in less advanced pancreatic tumor lesions may offer the opportunity to improve anti-tumor stasis and/or killing due to better target cell access. Here, we demonstrate the efficacy of amatuximab of single agent as well as in combination with gemcitabine against peritoneal metastatic pancreatic cancer under an adjuvant mouse model system.

## RESULTS

### Mesothelin expression across pancreatic cancer cell lines

**Figure 1:**
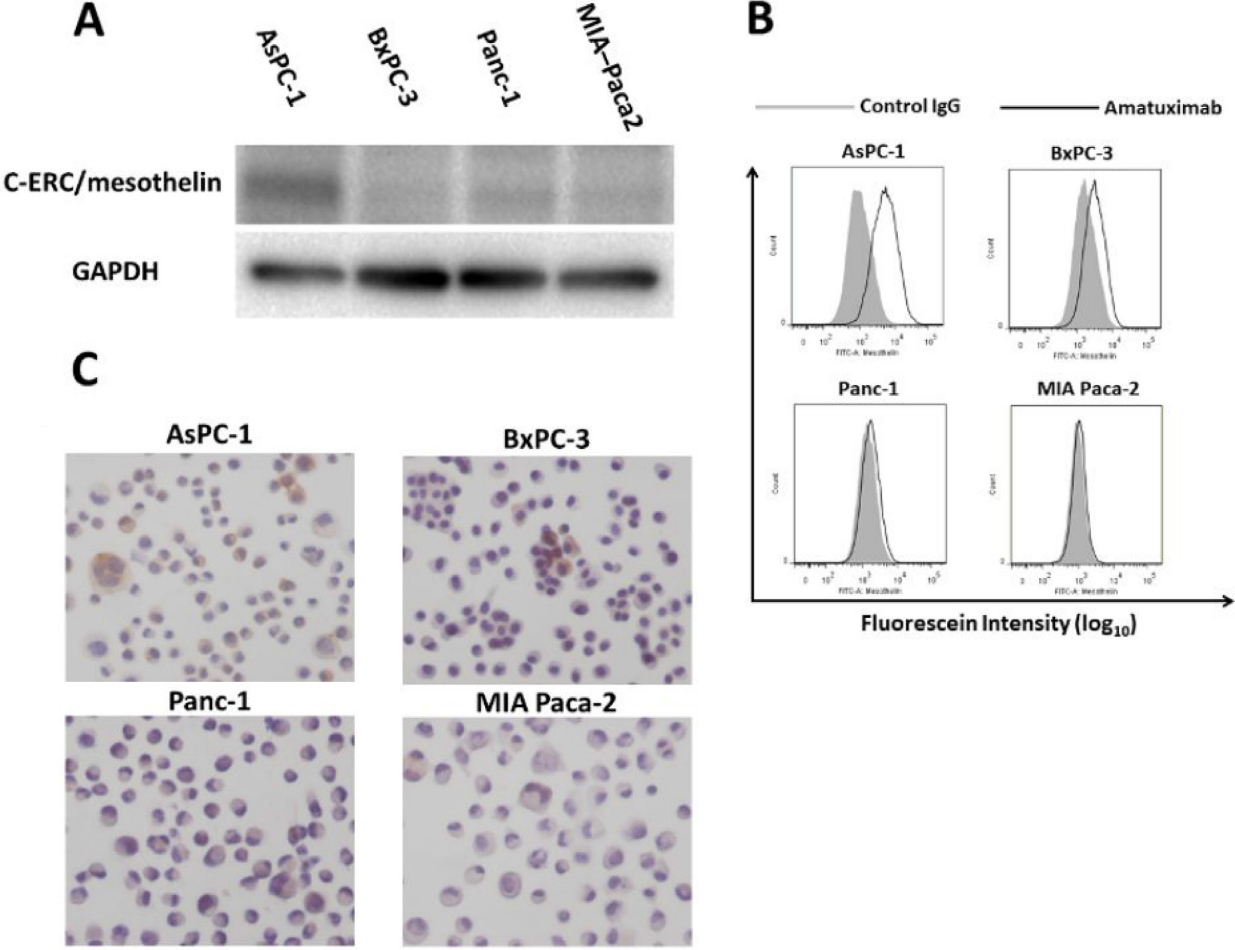
Comparison of mesothelin expression in human pancreatic cancer cell lines Whole cell lysates of AsPC-1, BxPC-3, Panc-1 and MIA Paca-2 cells were analyzed for mesothelin expression by western blotting (**A**) with GADPH was used as an internal control; FACS analysis (**B**) or immunocytochemistry (**C**).

To identify pancreatic cancer derived cell lines naturally expressing robust levels of mesothelin, we analyzed AsPC-1, BxPC-3, Panc-1 and MIA Paca-2 cell lines. Western blotting analysis revealed that AsPC-1 showed the strongest expression of mesothelin among these cell lines, whereas BxPC-3 and MIA Paca-2 showed weak expression of mesothelin (Figure 1A). Fluorescence activated cell sorting (FACS) analysis confirmed that AsPC-1 exhibited the strongest cell surface mesothelin expression (Figure 1B). Finally, AsPC-1 was confirmed by immunocytochemistry, to express robust levels of mesothelin (Figure 1C). Based on these three sets of analyses, we decided to use AsPC-1 pancreatic cancer cell line as our model system to test the effects of anti-cancer agents in *in vitro* and *in vivo* metastasis.

### Generation of AsPC-1 luciferase cells and *in vivo* characterization

**Figure 2:**
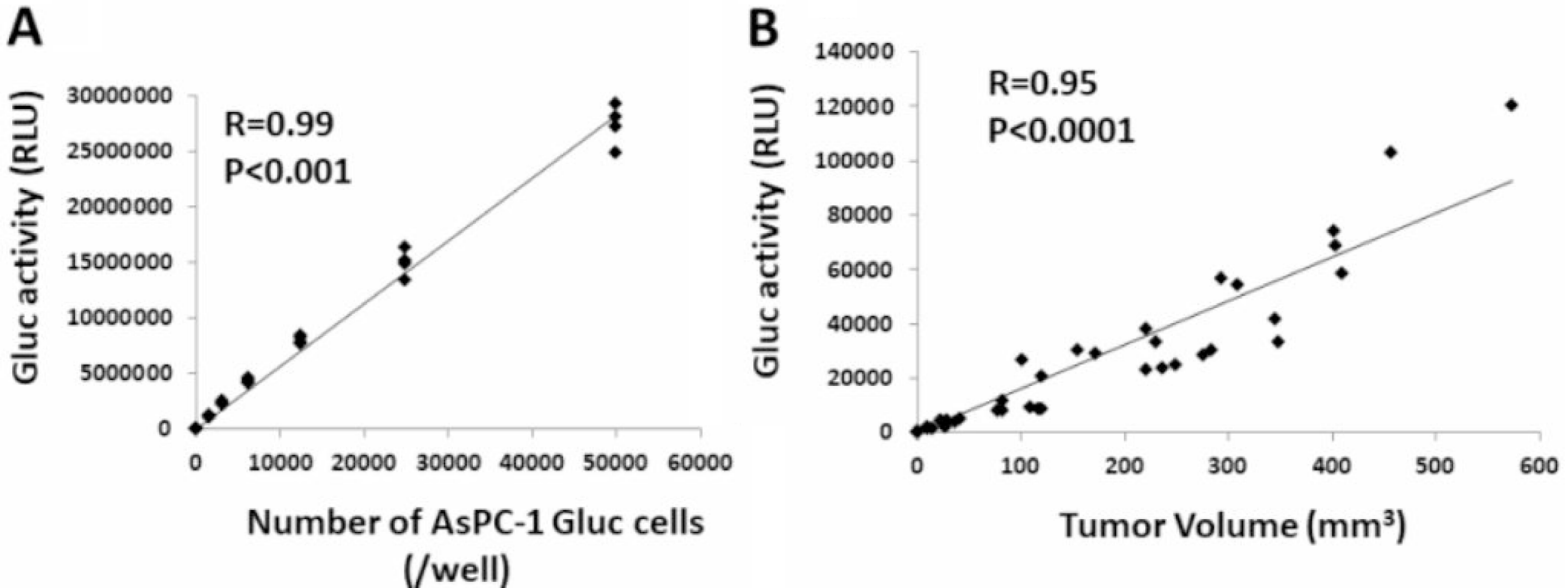
Establishment of luciferase-secreting pancreatic cancer cells and animal model (**A**) Correlation between the number of AsPC-1-Gluc cells and secreted Gluc activity *in vitro*. (**B**) Correlation between the tumor volume of AsPC-1-Gluc cells and secreted Gluc activity in a subcutaneous transplantation mouse model.

We established AsPC-1-Gluc cells, a pancreatic cancer cell line secreting luciferase as a model system to monitor tumor cell growth *in vivo* as described in the Methods section. Cells were injected into nude mice and analyzed for growth availability via luciferase activity. As shown in Figure 2A, *in vitro* the secreted Gluc activity and the number of cells showed a significant correlation (*p* < 0.05), demonstrating that the activity of secreted Gluc is a correlative marker for live cells. *In vivo*, the subcutaneous tumor size in each mouse strongly correlated with the signal intensity of plasma Gluc activity (*p* < 0.05, correlation coefficient: R = 0.95) (Figure 2B). These results suggest that AsPC-1-Gluc is a useful assay system to evaluate the tumor volume of PC cells *in vivo*.

### Amatuximab prevents the development of tumor mass in a peritoneal metastasis mouse model

**Figure 3:**
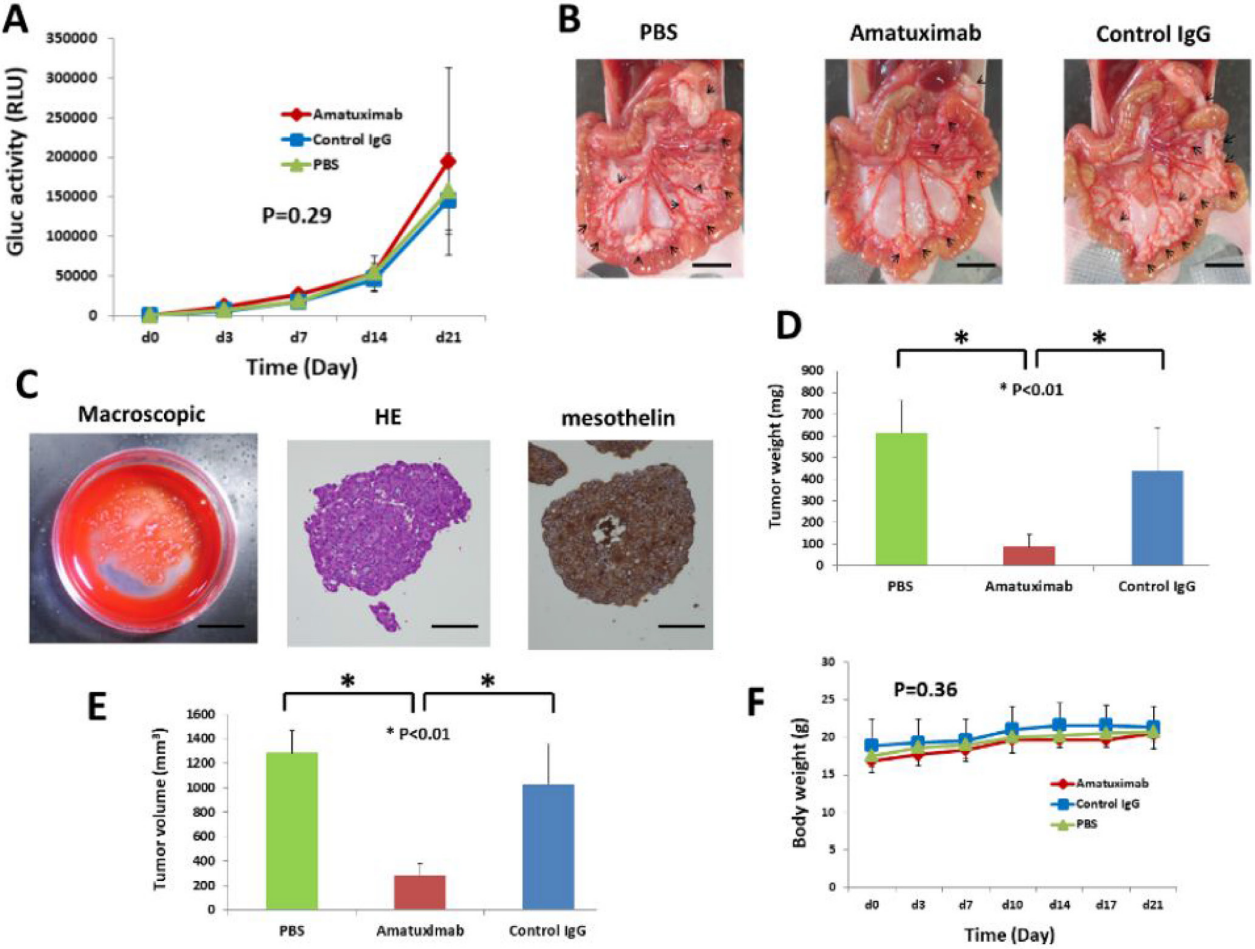
Amatuximab prevents the development of tumor masses in an AsPC-1-Gluc peritoneal metastasis mouse model (**A**) Time course of changes of Gluc activity in mouse plasma. Error bars represent the SEM. (**B**) Macroscopic (scale bar: 10 mm) findings of AsPC-1Gluc tumor dissemination in the mesenteries of nude mice. Arrows indicate tumor nodules. The result of a representative mouse is shown. (**C**) Findings of sherbet-like aggregations. Macroscopic (scale bar: 10 mm), HE staining (magnification: 400×, scale bar: 50 µm) and mesothelin staining (magnification: 400×, scale bar: 50 µm). Differences in final tumor weight (**D**) and tumor volume (**E**) among the three treatment groups. Error bars represent the SEM. (**F**) Body weight of mice in the three treatment groups. Error bars represent the SEM.

Based on the above results, showing a strong correlation between plasma Gluc activity and the number of pancreatic cancer cells, employed AsPC-1-Gluc cells in an *in vivo* study to evaluate whether amatuximab can affect peritoneal metastasis of PC. As shown in Figure 3A, no statistically significant difference was observed in any group, however, macroscopic analysis showed that amatuximab treated mice had a reduced number of gross tumor masses (Figure 3B) and developed sherbet-like aggregates (Figure 3C) in all cases, despite having not formed any aggregates in other groups (Table 1).

**Table 1:** Intraperitoneal findings in each group

	Control (PBS)	Amatuximab (200 mg/kg)	Control IgG (200 mg/kg)
**Ascites**	1/6	3/6	1/7
**Disseminated metastasis**			
Mesenterium	6/6	6/6	7/7
Omentum	6/6	6/6	7/7
Peritoneum	6/6	6/6	7/7
Diaphragm	3/6	0/6	3/7
Liver	1/6	0/6	0/7
Pancreas	6/6	6/6	7/7
Kidney	6/6	6/6	7/7
Spleen	6/6	6/6	7/7
**Lymph nodes metastasis**	6/6	6/6	7/7
**Sharbet like aggregates**	0/6	**6/6**	0/7

Moreover, the single-agent amatuximab-treated group showed significantly reduced tumor weight (*p* < 0.01) and volume (*p* < 0.01) as compared with control groups (Figure 3D, 3E). No reduction in body weight was observed in amatuximab treated mice supporting amatuximab’s tumor-specific targeting activity (Figure 3F).

### Amatuximab enhances the anti-tumor effects of GEM in a peritoneal metastasis mouse model

We showed in experiments above that while metastatic lesions were suppressed by single-agent amatuximab, overall tumor mass formation was not significantly impacted by amatuximab treatment. To determine if addition of a pancreatic cancer standard-of-care chemotherapeutic agent would have enhanced anti-metastatic effects by amatuximab on a pancreatic cancer peritoneal metastasis mouse model, we conducted additional *in vivo* studies testing amatuximab in combination with GEM.

**Figure 4:**
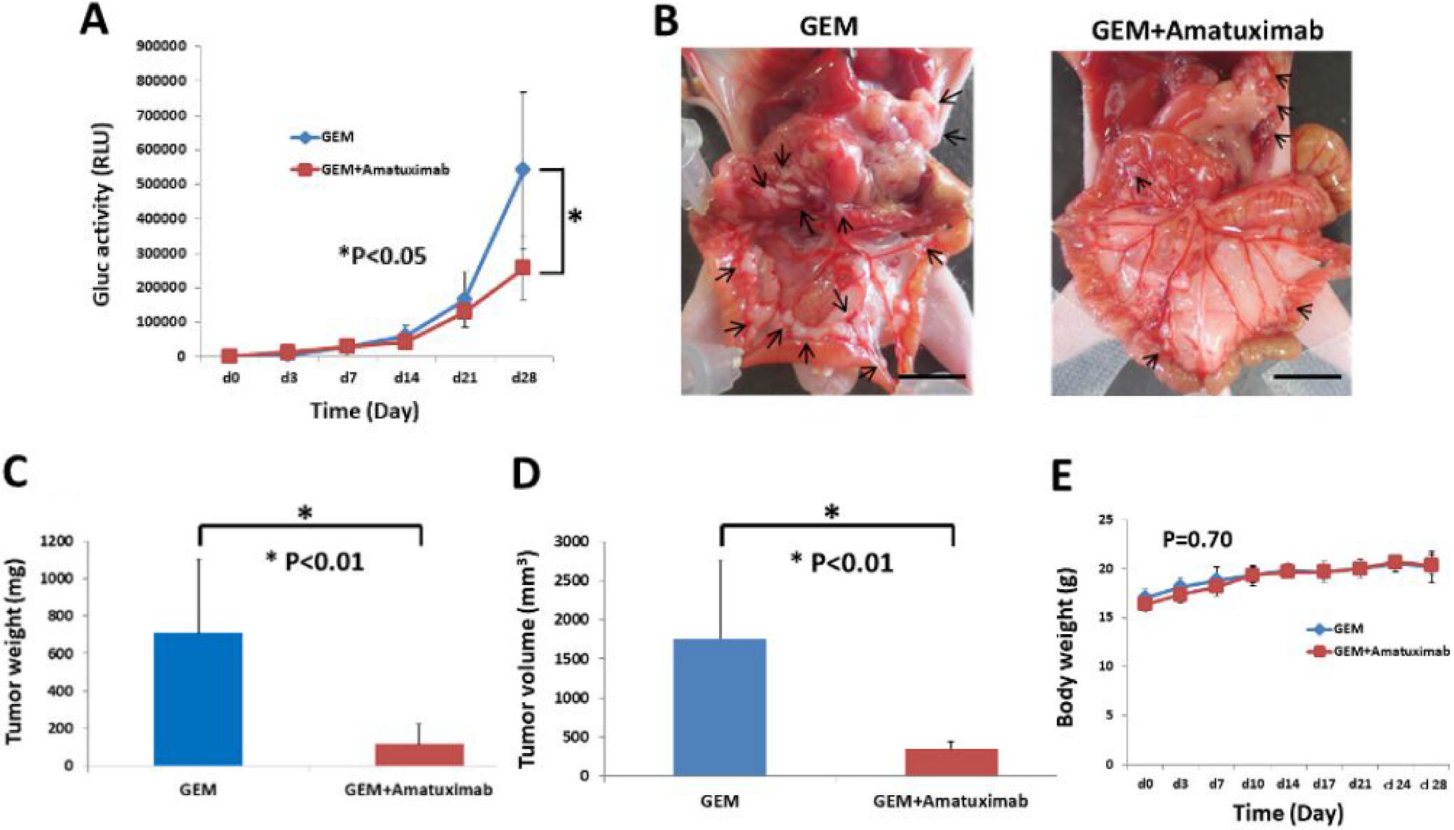
Combination therapy of GEM and amatuximab enhances anti-tumor effects against isolated cancer cells in an AsPC-1-Gluc peritoneal metastasis mouse model AsPC-1-Gluc cells (5 × 10^6^/mouse) were injected into the peritoneal cavities of nude mice. These mice were treated twice a day with an IP injection of GEM alone or GEM plus amatuximab (*n* = 6). (**A**) Time course of changes of Gluc activity in mouse plasma. Error bars represent the SEM. (**B**) Macroscopic (scale bar: 10 mm) findings of AsPC-1Gluc tumor dissemination in the mesenteries of nude mice. Arrows indicate tumor nodules. Differences in final tumor weight (**C**) and tumor volume (**D**) in the two treatment groups. Error bars represent the SEM. (**E**) Body weight of mice in the two treatment groups. Error bars represent the SEM.

As shown in Figure 4A, there was a significant difference in metastatic tumor growth at day 28 between the GEM alone and GEM plus amatuximab treated groups.

Macroscopic analysis showed that mice treated with amatuximab plus GEM had significantly reduced number of gross tumor masses as compared to GEM alone treated mice (Figure 4B) (Table 2). Furthermore, amatuximab plus GEM treated mice showed significant reductions in tumor weight (*p* < 0.01) and lesion volume (*p* < 0.01) as compared with the GEM monotherapy group (Figure 4C, 4D) while no significant changes in body weight were observed in either group (Figure 4E).

**Table 2:** Intraoperative findings in each treated group

	GEM	GEM+Amatuximab
**Ascites**	0/6	0/6
**Disseminated metastasis**		
Mesenterium	6/6	6/6
Omentum	6/6	6/6
Peritoneum	6/6	6/6
Diaphragm	5/6	2/6
Liver	1/6	0/6
Pancreas	6/6	6/6
Kidney	6/6	6/6
Spleen	6/6	6/6
**Lymph nodes metastasis**	6/6	6/6
**Sharbet like aggregates**	0/6	1/6

## DISCUSSION

Here, we demonstrated that the anti-mesothelin antibody amatuximab suppressed peritoneal metastases and enhanced the anti-tumor effects of gemcitabine in peritoneal metastases of AsPC-1-Gluc cells in a mouse model of pancreatic cancer.

This effect appeared as a result of amatuximab to preventing the development of tumor masses, reduction in tumor cell viability and overall gross tumor mass. Interestingly, sherbet-like aggregates, which are cancer cells that are biologically active but have not formed a gross tumor mass were only found in the amatuximab treated group. This effect is consistent with amatuximab’s anti-tumor activity by utilizing immune-effector cells to kill antibody-bound cells via ADCC as well as block heterotypic cell formation via blockage of mesothelin to its cognate receptor on neighboring cells. This effect was enhanced when amatuximab was used in combination with GEM.

Interestingly, a phase I clinical trial to evaluating the mechanisms of amatuximab in patients with mesothelin-expressing cancers reported an increase in serum CA125 levels in all patients with mesothelioma [16] and subsequently ovarian cancer that was attributed to amatuximab inhibiting the binding of cell associated mesothelin and CA125, thereby preventing the intraperitoneal / intrapleural spread of ovarian cancer and mesothelioma. These findings suggested that amatuximab might be most useful for the treatment of mesothelioma and ovarian cancer.

In pancreatic ductal carcinoma, Shimizu et al. [21] reported that CA125 and mesothelin bind and that the blockage of CA125 binding to mesothelin by an antibody inhibited both invasion and migration of a pancreatic cancer cell line *in vitro*.

Our findings further supported these observations *in vivo* whereby amatuximab suppressed the development of tumor masses.

This effect along with broader tumor cell killing can be further enhanced using amatuximab in combination with GEM, an agent approved for the treatment of pancreatic cancer. While our study did not uncover the exact mechanism of the synergy between these agents, our results suggest that amatuximab improves access of GEM to pancreatic cells by reducing their metastatic volume. Moreover our findings suggest that amatuximab might be best used in an adjuvant clinical setting testing its ability to suppress peritoneal dissemination of small volume or single pancreatic cancer cell lesions. Recently, post hoc analysis of amatuximab in a trial testing patients with primary diagnosed, unresectable malignant pleural mesothelioma found that patients treated in combination with amatuximab plus cisplatin/pemetrexed standard-of-care tended to show statistical improvement in progression free survival and overall survival in patients with low baseline serum CA125 levels in contrast to those with high levels [18]. These data suggest lower levels of mesothelin’s cognate receptor MUC16/CA125 can result in improved clinical outcome by amatuximab treatment, thereby supporting blocking its heterotypic function in part may improve anti-tumor activity.

## MATERIALS AND METHODS

### Cell culture

The human pancreatic cancer cell lines AsPC-1, BxPC-3, Panc-1 and MiaPaCa-2 were obtained from ATCC (Manassas, VA, USA). AsPC-1 was maintained in RPMI-1640 culture medium (Sigma-Aldrich, Tokyo, Japan) and BxPC-3, Panc-1 and MiaPaCa-2 were maintained in DMEM culture medium (Sigma-Aldrich). Each culture medium was supplemented with 10% FBS, 100 U/ml penicillin, and 100 µg/ml streptomycin. All cells were cultured in a humidified incubator containing 5% CO_2_ in air at 37°C.

### Reagents

Amatuximab (also known as MORAb-009) was obtained from MORPHOTEK Inc. (Exton, USA). GEM was purchased from Eli Lilly (Indianapolis, USA).

### Western blot analysis and antibodies

Cells were harvested after treatment and washed, then homogenized at 4°C in lysis buffer (0.1% SDS, 1% Igepal CA-630, 0.5% sodium deoxycholate) and a protease inhibitor cocktail (Sigma-Aldrich, St. Louis, MO, USA). Cell lysates (20–50 µg) were resolved by electrophoresis on polyacrylamide gels and transferred to PVDF membranes (Millipore, Billerica, MA, USA). After blocking the membranes in 5% non-fat dry milk or 3% bovine serum albumin in Tris-buffered saline for 1 h at room temp, the blots were hybridized overnight at 4°C with primary antibodies. After hybridization with secondary antibodies conjugated with HRP (Cell Signaling Technology, Danvers, MA, USA), immunocomplexes were visualized using an enhanced chemiluminescence kit (GE Healthcare, Chalfont St. Giles, UK). Primary antibodies included antibodies against mesothelin (Immuno-Biological Laboratories, Fukuoka, Japan) and GAPDH (Cell Signaling Technology, Danvers, MA, USA). All primary antibodies were diluted at 1:2000.

### FACS analysis

Cultured cells were washed and resuspended in ice-cold PBS. Primary antibody was added at 2 µg/ml and incubated for 1 h on ice. Cells were then washed with PBS, incubated with Alexa Fluor 488 goat-anti-mouse secondary antibody (Invitrogen, Carlsbad, CA, USA) diluted to 1 µg/ml, washed as above and analyzed on BD FACS Canto™ II flow cytometer (BD Biosciences).

### Immunohistochemistry

Immunohistochemistry was performed using the EnVision+ System-HRP (Dako Japan, Tokyo). Briefly, the sections were mounted on charged glass slides, deparaffinized, and rehydrated through a graded ethanol series. Antigens were retrieved in Dako EnVision FLEX Target Retrieval Solution low pH using Dako PT Link for 20 min at 97°C according to the manufacturer’s instructions (Dako, Japan). After the blocking of endogenous peroxidase activity with 0.03% hydrogen peroxide, the tissue sections were incubated with a mouse monoclonal antibody against mesothelin (clone 5B2, Novocastra, Newcastle Upon Tyne, UK) at room temp for 30 min and then reacted with a dextran polymer reagent combined with secondary antibodies and peroxidase for 30 min at room temp. Specific antigen-antibody reactions were visualized with diaminobenzidine chromogen applied for 10 min. Slides were counterstained with hematoxylin, dehydrated and mounted.

### Animals

Six-week-old female BALB/c nu/nu mice were purchased from CLEA Japan, Inc. (Tokyo, Japan). Mice were maintained under specific pathogen-free conditions in laminar-flow benches and were allowed to adapt to the environment for 1 or 2 weeks before experiments. All procedures involving animals and their care were approved by the Ethics Committee of Hokkaido University and were conducted under institutional and Japanese governmental guidelines for animal experiments.

### Establishment of AsPC-1-Gluc and substrate preparation

For the quantitative evaluation of tumor volume and dissemination *in vivo*, we engineered a Gaussia luciferase (Gluc), a secretory form of luciferase isolated from the marine copepod, *Gaussia princeps*, expression vector into AsPC-1 cells. The vector carrying cassettes expressing a Gluc gene with a CMV promoter and a neomycin resistance gene with an SV40 promoter (New England BioLabs, Ipswich, MA, USA) was linearized with the restriction enzymes *Ssp*I and *Pci*l. AsPC-1 cells were transfected with this vector using FuGENE HD Transfection Reagent (Roche, Basel, Switzerland) according to the manufacturer’s instructions. Stably transfected cells were selected in 400 µg/ml G418 (Sigma) for approximately 3 weeks, cloned with cloning rings, and expanded to create monoclonal cell lines. The established cell line, AsPC-1-Gluc, was cultured in RPMI-1640 supplemented with 10% FBS, 100 U/ml penicillin, 100 µg/ml streptomycin, and 100 µg/ml G418. Substrate of BioLux Gaussia Luciferase Assay Kit (New England BioLabs, Ipswich, MA, USA) was used for *in vitro* or *ex vivo* experiments according to the manufacturer’s instructions.

### Correlation between the number of AsPC-1-Gluc cells and secreted Gluc activity

AsPC-1-Gluc cells were seeded in a 96-well microplate at varying cell densities (*n* = 4) and incubated overnight. Subsequently, 20 µl of the supernatant was mixed with 50 µl of the reaction buffer and chemiluminescence was measured for 2 sec using a Varioskan Flash microplate reader (Thermo Fisher Scientific, Waltham, MA, USA).

### Subcutaneous xenograft mouse model and correlation between tumor volume and Gluc activity in mouse plasma

AsPC-1-Gluc cells (5 × 10^6^) were harvested from subconfluent cultures, washed with PBS, resuspended in a mixture of 50 µl of PBS and 50 µl of Matrigel (growth factor reduced; BD Biosciences, Bedford, MA, USA), and subcutaneously injected into the right flank of nude mice (*n* = 5). A small amount of blood was collected from the tail vein with heparinized microhematocrit capillary tubes every 3 or 4 days and centrifuged at 12,000 rpm for 5 min. Subsequently, 5 µl of plasma was mixed with 50 µl of the reaction buffer and chemiluminescence was measured using a Varioskan Flash microplate reader as described above (Gluc plasma assay). The size of the subcutaneous tumor was measured at the time of blood draw. The following formula was used to calculate tumor volumes: tumor volume = (shorter diameter)^2^ × (longer diameter) × 0.5.

### Evaluation of the effect of amatuximab under an adjuvant setting in a peritoneal metastasis pancreatic cancer mouse model

AsPC-1-Gluc cells (5 × 10^6^)/250 µl RPMI-1640 medium were transplanted to the peritoneal cavity of nude mice on day 0. Next, PBS, amatuximab (200 mg/kg) or isotype control IgG (200 mg/kg) was intraperitoneally injected into nude mice twice a day, immediately after tumor cell inoculation. Each treatment group included six or seven mice.

Tumor volume was assessed by Gluc plasma assay on days 3, 7, 14 and 21. On day 22, mice were sacrificed (Supplementary Figure 1). Tumor tissue samples and sherbet-like aggregations were harvested and the total volume of disseminated tumor nodules was measured as described above. These collected specimens were fixed with formalin for 6 hours immediately after harvest, embedded in paraffin, sliced sequentially at a thickness of 3 µm and used for H&E staining or immunohistochemistry.

### Evaluation of the synergistic effect of amatuximab and GEM under an adjuvant setting in a peritoneal metastasis pancreatic cancer mouse model

AsPC-1-Gluc cells (5 × 10^6^) were washed with PBS, resuspended in 250 µl of RPMI-1640, and injected into nude mice intraperitoneally (*n* = 6). PBS (as control) or amatuximab (200 mg/kg) was intraperitoneally injected into nude mice twice a day until day 20. GEM (80 mg/kg) was administered to both groups on day 10, 17, 24. Tumor volume was assessed by Gluc plasma assay on days 3, 7, 14, 21 and 28. Mice were sacrificed at day 29 (Supplementary Figure 2), tumor tissue samples were harvested and whole tumor volume and weight was measured according to the formula described above.

### Statistical analysis

Correlations between two variables were evaluated with Pearson correlation coefficient. Differences between two groups were assessed using Student’s *t*-test. Multiple group comparisons were performed using one-way ANOVA with a post hoc test, Dunnett’s test, or Tukey–Kramer test for subsequent individual group comparisons. Comparisons between sequential data sets, such as Gluc activities of different treatment groups, were performed using one-way repeated measures ANOVA. A value of *p* < 0.05 was considered significant.

## SUPPLEMENTARY MATERIALS FIGURES


