# Supplementary material for: The anti-mesothelin monoclonal antibody amatuximab enhances the anti-tumor effect of gemcitabine against mesothelin-high expressing pancreatic cancer cells in a peritoneal metastasis mouse model
Source: Oncotarget. 2018 Sep 18;9(73):33844–52. doi: 10.18632/oncotarget.26117 (PMC6173461; doi:10.18632/oncotarget.26117)
Supplement: Supplementary file 1 [file oncotarget-09-33844-s001.pdf]

The anti-mesothelin monoclonal antibody amatuximab enhances the anti-tumor effect of gemcitabine against mesothelin-high expressing pancreatic cancer cells in a peritoneal metastasis mouse model

SUPPLEMENTARY MATERIALS

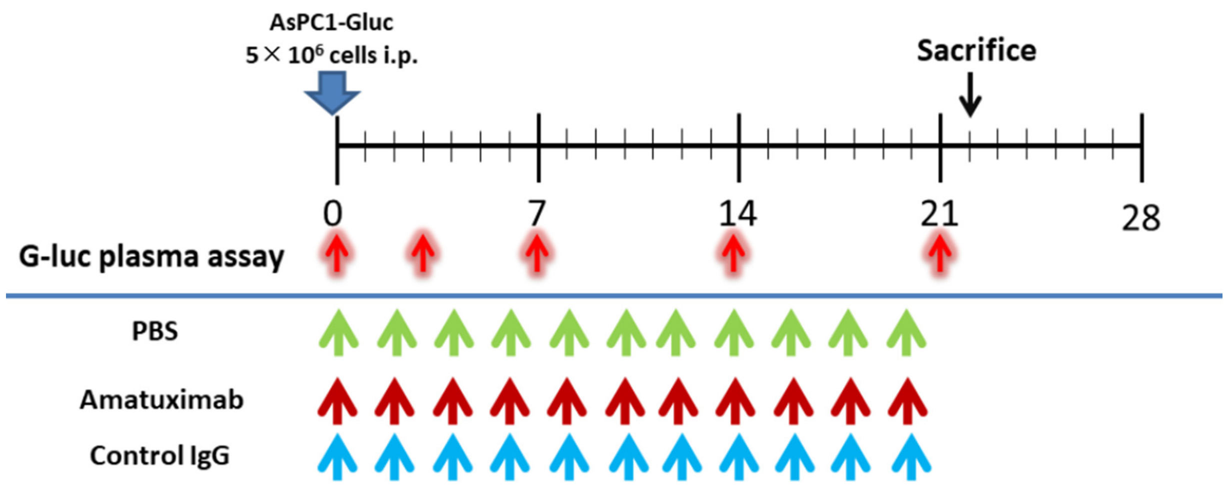

Supplementary Figure 1: Time schedule of evaluation of the effect of amatuximab under an adjuvant setting in a peritoneal metastasis pancreatic cancer mouse model.

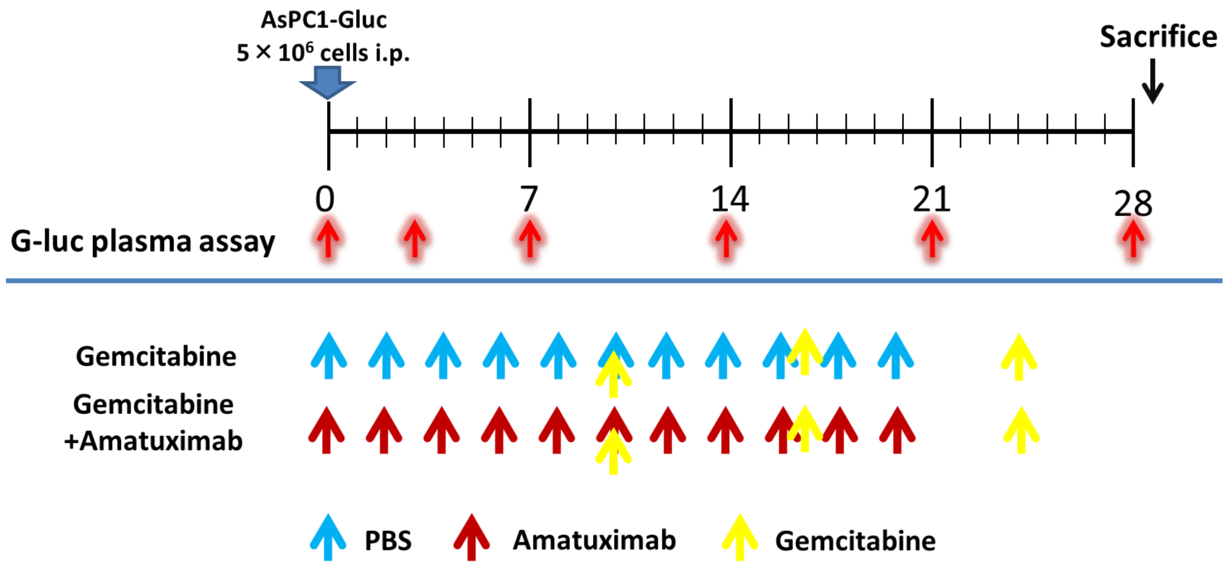

Supplementary Figure 2: Time schedule of combination therapy of GEM and amatuximab under an adjuvant setting in a peritoneal metastasis pancreatic cancer mouse model.
